# Early recovery of urinary continence after robot‐assisted radical prostatectomy is associated with membranous urethra and neurovascular bundle preservation

**DOI:** 10.1111/iju.15388

**Published:** 2024-01-09

**Authors:** Satoshi Ando, Toru Sugihara, Shiro Hinotsu, Hiroto Kishino, Daichi Hirata, Risako Watanabe, Atsushi Yanase, Hirotaka Yokoyama, Hayato Hoshina, Kaori Endo, Jun Kamei, Eiichiro Takaoka, Tetsuya Fujimura

**Affiliations:** ^1^ Department of Urology Jichi Medical University Shimotsuke Tochigi Japan; ^2^ Biostatistics and Data Management Sapporo Medical University Sapporo Hokkaido Japan

**Keywords:** prostate neoplasm, robotic surgical procedures, urinary incontinence

## Abstract

**Objectives:**

We investigated the correlation between surgical outcomes and postoperative urinary continence recovery in robot‐assisted radical prostatectomy (RARP).

**Methods:**

Patients who underwent RARP in our institution (*n* = 195) were included in this study. Preserved urethral length (PUL) was assessed during the procedure. Other outcomes of the surgical procedure were collected from operative records. Kaplan–Meier analysis with log‐rank test was used to compare urinary continence recovery rate with the PUL, sparing of the neurovascular bundle (NVB), and other surgical procedures. Univariate and multivariate analyses were performed using Cox proportional hazards model, and *p*‐values of <0.05 were considered significant.

**Results:**

Patients with a PUL ≥26 mm had 10.0%, 24.7%, 36.6%, and 89.0% continence recovery rates at 30, 60, 90, and 365 days after surgery, respectively, while patients with a PUL <26 mm had 0%, 17.8%, 26.1%, and 80.9% recovery rates, respectively. Kaplan–Meier curves showed significantly better postoperative urinary continence recovery at 30 days after RARP in patients with a PUL ≥26 mm than those with a PUL <26 mm (*p* = 0.0028) and in patients with NVB preservation than those with no NVB preservation (*p* = 0.014). Urinary continence recovery within 30, 60, and 90 days after surgery was 90.6% for patients with a PUL of ≥26 mm and NVB preservation, while only 82.3% for patients with a PUL of <26 mm or no NVB preservation.

**Conclusion:**

Our results suggest that a PUL ≥26 mm and NVB preservation after RARP correlate with a significantly higher postoperative rate of recovery of urinary continence.

**Abbreviations & Acronyms**
BMI: body mass index
IRB: institutional review board
MRI: magnetic resonance imaging
MUL: membranous urethral length
NVB: neurovascular bundle
PSA: prostate‐specific antigen
PSM: positive surgical margin
PUL: preserved urethral length
RARP: robot‐assisted radical prostatectomy
ROC: receiver operating characteristic

## INTRODUCTION

In 2018, 92 021 men in Japan developed prostate cancer, the highest among all carcinomas^1^ and the sixth highest cause of cancer deaths in Japan in 2020 (12 759 deaths).^2^

A common treatment for localized and locally advanced prostate cancer is robot‐assisted radical prostatectomy (RARP). However, after surgery, urinary incontinence can persist and reduce quality of life by affecting physical activity, social relationships, emotional health, ability to travel, anxiety levels, and depression.^3^ Therefore, urinary incontinence after RARP is a serious concern.

Factors associated with postoperative incontinence include patient selection, pre‐ and postoperative membranous urethral length (MUL), and intraoperative procedures affecting the cavernous nerve of the penis, fascia, and ligaments. In addition, the postoperative duration of urinary catheter placement has been mentioned as associated with postoperative incontinence in previous reports.^4^ Further, a study revealed that sympathetic, sensory, and nitrergic nerves are distributed in the internal and external urethral sphincter along with autonomic fibers within the neurovascular bundles (NVBs).^5^ The authors concluded that during radical prostatectomy, the NVBs should be preserved.^5^ Although various factors may affect postoperative urinary incontinence, a definitive factor has not been determined.

In our previous study, preserved urethral length (PUL) after RARP was found to correlate significantly with postoperative urinary continence recovery rate.^6^ In this study, we measured the PUL quantitatively during RARP and investigated the association of both PUL and NVB preservation with postoperative urinary continence recovery.

## PATIENTS AND METHODS

This study was approved by our institute's institutional review board (IRB) (IRB number: A19‐199). Patients who underwent RARP for prostate cancer at our institution between May 2020 and March 2022 were enrolled in the study.

Patient characteristics, perioperative outcomes, and pathological findings, including age, body mass index (BMI), preoperative prostate‐specific antigen (PSA), date of surgery, presence of urinary continence after RARP, number of days to achieve urinary continence, date of the last consultation, total operative time, console time, loss of blood volume, weight of the resected prostate, pathological T stage, surgical margin status, and follow‐up periods, were obtained from medical records, as described previously.^6^ Continence recovery was indicated when social continence was attained (use of a small 20‐mL pad required daily), as described previously.^6^ On postoperative day 6, urethral catheters were removed, and patients were instructed to perform pelvic floor exercises postoperatively.

Operative records and case summaries revealed surgical skill outcomes, such as preservation of NVBs, the puboprostatic ligament, endopelvic fascia, and PUL.

### Surgical techniques of RARP

**FIGURE 1:**
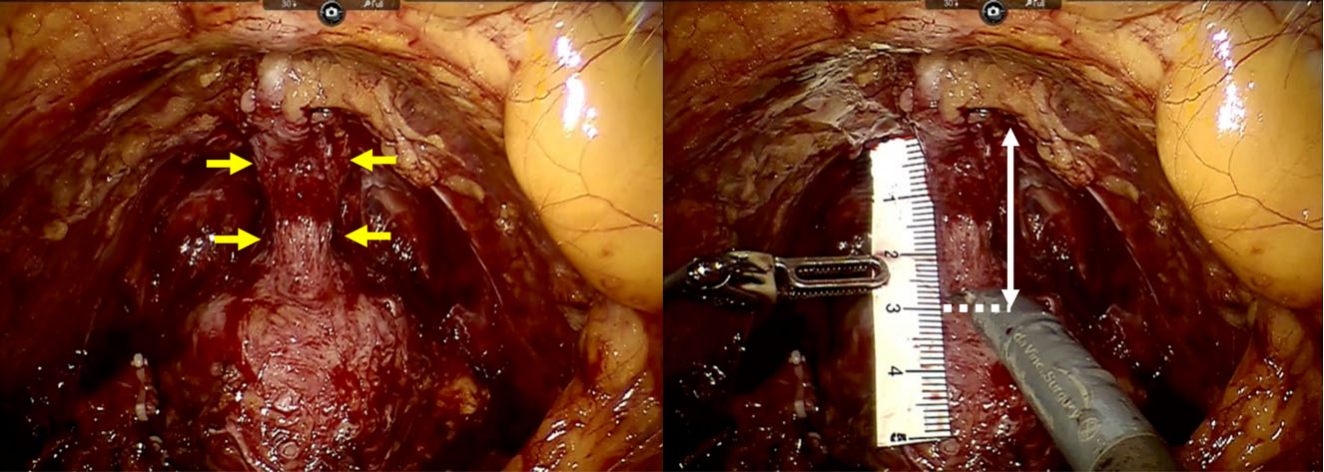
Measurement of the preserved urethral length (PUL). The white arrow indicates urethral length. In this example, the PUL is 28 mm. The yellow arrows indicate the urethra.

Transperitoneal RARP with six ports was performed, as described previously.^6^ Magnetic resonance imaging (MRI) findings and the positive prostate biopsy site indicated the sparing side for the NVB. Bilateral NVB were spared when the positive prostate biopsy site was in the transition zone. The PUL was measured using a ruler intracorporeally prior to incision of the urethra, with the prostate pulled superiorly (Figure 1). Only posterior reconstruction was performed. RARP was performed by 9 surgeons as an operator. One surgeon (T.F.) also participated in all of the procedures as an instructive assistant.

### Statistical analyses

Continuous data were subjected to the Wilcoxon rank sum test, while the Chi‐squared test was used for categorical data. Continence recovery rate curves were plotted. We used the Kaplan–Meier method to plot continence recovery rate curves, with comparisons using the log‐rank test. The correlation between clinical parameters and continence recovery was examined using univariate and multivariate Cox proportional hazards models, with median values used to determine cut‐offs for age, BMI, and resected prostatic volume. The cut‐off value for PUL was determined by receiver operating characteristic (ROC) curves. All statistical analyses were performed using the JMP Pro version 16.0.0 software (SAS Institute Inc., Cary, NC, USA), with a *p*‐value of 0.05 considered to represent a significant difference.

## RESULTS

Characteristics and perioperative findings of the 195 patients enrolled in the study are summarized in Table 1. Of these patients, 21, 107, and 67 patients had bilateral, unilateral, and non‐NVB‐sparing operations, respectively, and 147, 29, and 19 patients had bilateral, unilateral, and non‐fascia‐sparing operations, respectively. In addition, 156, 28, and 11 patients had bilateral, unilateral, and non‐puboprostatic ligament‐sparing operations, respectively. The median PUL was 27 mm. The median postoperative follow‐up period was 381 days (interquartile range, 292–528 days). Table 2 shows a summary of oncological data and postoperative recurrence. An ROC curve determined the cut‐off value for PUL as 26 mm (Figure S1).

**TABLE 1:** Characteristics and perioperative parameters of the patients.

	All	PUL ≥ 26 mm	PUL < 26 mm	*p*‐Value	NVB (+)	NVB (−)	*p*‐Value	PUL ≥ 26 mm and NVB (+)	PUL < 26 mm or NVB (−)	*p*‐Value
	*n* = 195	*n* = 110	*n* = 85		*n* = 128	*n* = 67		*n* = 74	*n* = 121	
Age, years, median (IQR)	69 (63–73)	68 (63–73)	69 (63–73.5)	0.60	68 (63–73)	71 (65–74)	0.071	68 (63–73)	70 (64–74)	0.21
BMI, median (IQR)	24.2 (22.8–26.3)	24.1 (22.9–26.2)	24.2 (22.3–26.3)	0.61	23.9 (22.7–26.1)	24.5 (22.8–26.5)	0.24	23.9 (22.9–26.2)	24.2 (22.4–26.4)	0.72
Preoperative PSA, ng/mL, median (IQR)	7.3 (5.6–11.7)	7.9 (6.0–15.2)	7.9 (6.0–15.2)	0.022	7.0 (5.2–10.1)	8.3 (6.0–17.4)	0.011	6.6 (4.7–9.6)	7.9 (5.9–14.3)	0.010
Resected prostate weight, g, median (IQR)	39.3 (32.1–48.8)	40.2 (34.6–51.7)	37.5 (28.8–45.3)	0.022	39.2 (32–50.1)	39.4 (34.1–46.3)	1.00	40.3 (33.9–51.8)	38.9 (30.2–46.4)	0.37
Operative time, min, median (IQR)	177 (141–172)	170 (143–211)	196 (151–230)	0.0029	171 (141–212)	180 (149–235)	0.073	157 (134–205)	184 (149–229)	0.0009
Console time, min, median (IQR)	129 (103–172)	120 (97–168)	145 (109–185)	0.0033	120 (97.3–164.8)	140 (108–198)	0.052	113 (93–153)	142 (108–185)	0.0007
Bleeding loss, mL, median (IQR)	200 (100–300)	150 (100–300)	200 (100–300)	0.50	200 (100–300)	200 (100–370)	0.60	150 (100–250)	200 (100–305)	0.13
NVB sparing (%)	Uni 107 (54.9)	Uni 53 (48.2)	Uni43 (50.6)		Uni107 (83.6)	No 67 (100)		Uni 64 (86.5)	Uni 43 (35.5)	< 0.0001
	Bil 21 (10.8)	Bil 9 (8.2)	Bil 11 (12.9)	0.49			<0.0001		Bil 11 (9.1)	
	No 67 (34.4)	No 23 (20.9)	No 31 (36.5)		Bil 21 (16.4)			Bil 10 (13.5)	No 67 (55.4)	
Endopelvic fascia sparing (%)	Uni 29 (14.9)	Uni 9 (8.2)	Uni 16 (18.8)		Uni22 (17.2)	Uni 7 (10.4)	<0.0001	Uni 12 (16.2)	Uni 17 (14.0)	0.0047
	Bil 147 (75.4)	Bil 68 (61.2)	Bil 60 (70.6)	0.34	Bil 103 (80.5)	Bil 44 (65.7)		Bil 61 (82.4)	Bil 86 (71.1)	
	No 19 (9.7)	No 8 (7.3)	No 9 (10.6)		No 3 (2.3)	No 16 (23.9)		No 1 (1.4)	No 18 (14.9)	
Puboprostatic ligament‐sparing (%)	Uni 28 (14.4)	Uni 13 (11.8)	Uni 12 (14.1)		Uni 18 (14.0)	Uni 10 (14.9)	0.0029	Uni 12 (16.2)	Uni 18 (14.9)	0.38
	Bil 156 (80.0)	Bil 66 (60.0)	Bil 69 (81.2)	0.92	Bil 108 (84.4)	Bil 48 (6.0)		Bil 62 (83.8)	Bil 94 (77.7)	
	No 11 (5.6)	No 6 (2.7)	No 4 (4.7)		No 2 (1.6)	No 9 (13.4)		No 2 (2.7)	No 9 (7.4)	
PUL, mm, median (IQR)	27 (24–32)	30 (30–33.5)	23.5 (22–25)	< 0.0001	28 (24–31.3)	26 (22.5–31)	0.49	30 (28–32.8)	25 (22–28)	< 0.0001

Abbreviations: Bil, bilateral; BMI, body mass index; IQR, interquartile range; NVB, neurovascular bundle; PSA, prostate‐specific antigen; PUL, preserved urethral length; Uni, unilateral.

**TABLE 2:** Pathological findings and postoperative recurrence.

	All patients	Patients with PUL ≥26 mm	Patients with PUL <26 mm	Patients with NVB (+)	Patients with NVB (−)	Patients with PUL ≥26 mm and NVB (+)	Patients with PUL <26 mm or NVB (−)
	*n* = 195	*n* = 110	*n* = 85	*n* = 128	*n* = 67	*n* = 74	*n* = 121
Biopsy ISUP grade 1, *n* (%)	18 (9.2)	13 (11.8)	5 (5.9)	14 (10.9)	4 (6.0)	11 (14.9)	7 (5.8)
Grade 2, *n* (%)	54 (27.7)	31 (28.2)	23 (27.1)	41 (32.0)	13 (19.4)	25 (33.8)	29 (24.0)
Grade 3, *n* (%)	42 (21.5)	24 (21.8)	18 (21.2)	28 (21.9)	14 (20.9)	16 (21.6)	26 (21.5)
Grade 4, *n* (%)	39 (20.0)	23 (20.9)	16 (18.8)	23 (18.0)	16 (23.9)	13 (17.6)	26 (21.5)
Grade 5, *n* (%)	42 (21.5)	19 (17.3)	23 (27.1)	22 (17.2)	20 (30.0)	9 (12.2)	33 (27.3)
Pathological T stage, *n* (%)							
pT2a	14 (7.2)	7 (6.4)	7 (8.2)	13 (10.2)	1 (1.5)	7 (9.5)	7 (5.8)
pT2b	4 (2.1)	3 (52.7)	1 (1.2)	3 (23.4)	1 (1.5)	2 (2.7)	2 (9.5)
pT2c	96 (49.2)	58 (16.4)	38 (44.7)	70 (54.7)	26 (38.8)	52 (70.3)	44 (36.4)
pT3a	38 (19.5)	18 (16.4)	20 (23.5)	19 (14.8)	19 (28.4)	29 (39.2)	9 (7.4)
pT3b	33 (16.9)	20 (18.2)	13 (15.3)	18 (14.1)	15 (22.4)	23 (31.1)	10 (8.3)
pT4	1 (0.5)	0	1 (1.2)	0	1 (1.5)	1 (1.4)	0
pT2+	1 (0.5)	1 (0.9)	0	1 (0.8)	0	0	1 (0.8)
pT2a+	1 (0.5)	0	1 (1.2)	1 (0.8)	0	1 (1.4)	0
pT2c+	2 (1.0)	0	2 (2.4)	1 (0.8)	1 (1.5)	2 (2.7)	0
ypT2a	1 (1.0)	1 (0.9)	0	1 (0.8)	0	0	1 (0.8)
ypT2c	2(1.0)	1 (0.9)	1 (1.2)	0	2 (3.0)	2 (2.7)	0
pTX	2(1.0)	1 (0.9)	1 (1.2)	1 (0.8)	1 (1.5)	2 (2.7)	0
Positive surgical margins, *n* (%)							
All stages, *n* (%)	73 (37.4)	40 (36.4)	33 (38.8)	31 (24.2)	42 (62.7)	24 (32.4)	48 (39.7)
pT2, *n* (%)	33 (16.9)	19 (17.3)	14 (16.5)	10 (7.8)	44 (65.7)	52 (70.3)	61 (50.4)
pT3, *n* (%)	35 (17.9)	20 (18.2)	15 (17.6)	21 (16.4)	17 (25.4)	19 (25.7)	52 (43.0)
pT2+, *n* (%)	4 (2.1)	1 (0.9)	3 (3.5)	1 (0.8)	2 (3.0)	1 (1.4)	3 (2.5)

Abbreviations: ISUP, International Society of Urological Pathology; NVB, neurovascular bundle; PUL, preserved urethral length.

Age, BMI, NVB preservation, endopelvic fascia preservation, puboprostatic ligament preservation, PUL, and resected prostatic volume were examined in univariate and multivariate analyses using the Cox proportional hazards model. Among all patients, no significant difference in postoperative urinary continence recovery at 30, 60, 90, and 365 days after RARP was observed. Univariate and multivariate analyses of NVB preservation, endopelvic fascia preservation, puboprostatic ligament preservation, and PUL at 30 days after RARP were not evaluated as no events occurred (Table 3).

**TABLE 3:** Cox proportional hazards model at 30, 60, 90, and 365 days postoperatively.

	Univariate												Multivariate											
	30 days			60 days			90 days			365 days			30 days			60 days			90 days			365 days		
	HR	95% CI	*p* Value	HR	95% CI	*p* Value	HR	95% CI	*p* Value	HR	95% CI	*p* Value	HR	95% CI	*p* Value	HR	95% CI	*p* Value	HR	95% CI	*p* Value	HR	95% CI	*p* Value
Age (≥69 vs. <69)	1.1	0.34–3.6	0.87	0.68	0.37–1.26	0.22	0.84	0.51–1.39	0.5	0.96	0.70–1.31	0.8	1.00	0.30–3.36	0.99	0.72	0.38–1.35	0.3	0.83	0.50–1.39	0.48	1.02	0.74–1.42	0.9
BMI (≥24.2 vs. <24.2)	0.59	0.17–2.0	0.39	0.87	0.47–1.60	0.65	0.74	0.45–1.23	0.24	1.14	0.83–1.55	0.42	0.55	0.15–1.98	0.36	0.83	0.44–1.58	0.57	0.7	0.41–1.19	0.18	1.18	0.84–1.66	0.34
Preservation of NVB (yes vs. no)	NE	NE	NE	1.6	0.81–3.19	0.16	1.4	0.81–2.42	0.22	1.23	0.89–1.71	0.2	NE	NE	NE	1.73	0.79–3.80	0.16	1.4	0.76–2.61	0.27	1.24	0.86–1.77	0.24
Preservation of endopelvic fascia (yes vs. no)	NE	NE	NE	0.82	0.32–2.09	0.68	0.86	0.40–1.88	0.7	1.25	0.74–2.13	0.39	NE	NE	NE	0.65	0.22–1.95	0.46	0.76	0.31–1.88	0.56	1.03	0.56–1.89	0.91
Preservation of puboprostatic ligament (yes vs. no)	NE	NE	NE	0.79	0.69–2.55	0.7	1.19	0.37–3.78	0.77	1.3	0.66–2.54	0.43	NE	NE	NE	0.77	0.22–2.75	0.7	1.23	0.37–4.11	0.73	1.24	0.86–2.63	0.48
PUL (≥26 vs. <26 mm)	NE	NE	NE	1.5	0.80–2.81	0.2	1.52	0.91–2.57	0.11	1.24	0.91–1.70	0.17	NE	NE	NE	1.48	0.78–2.80	0.22	1.48	0.88–2.50	0.14	1.27	0.92–1.75	0.13
Resected prostatic volume (≥39.3 vs. <39.3)	1.2	0.37–4.0	0.75	1.14	0.62–2.09	0.67	1.37	0.83–2.26	0.22	1.02	0.75–1.39	0.91	1.38	0.41–4.66	0.60	1.07	0/57–2.03	0.82	1.4	0.82–2.39	0.21	0.99	0.71–1.38	0.95

Abbreviations: BMI, body mass index; NE, not evaluated; NVB, neurovascular bundle; PUL, preserved urethral length.

**FIGURE 2:**
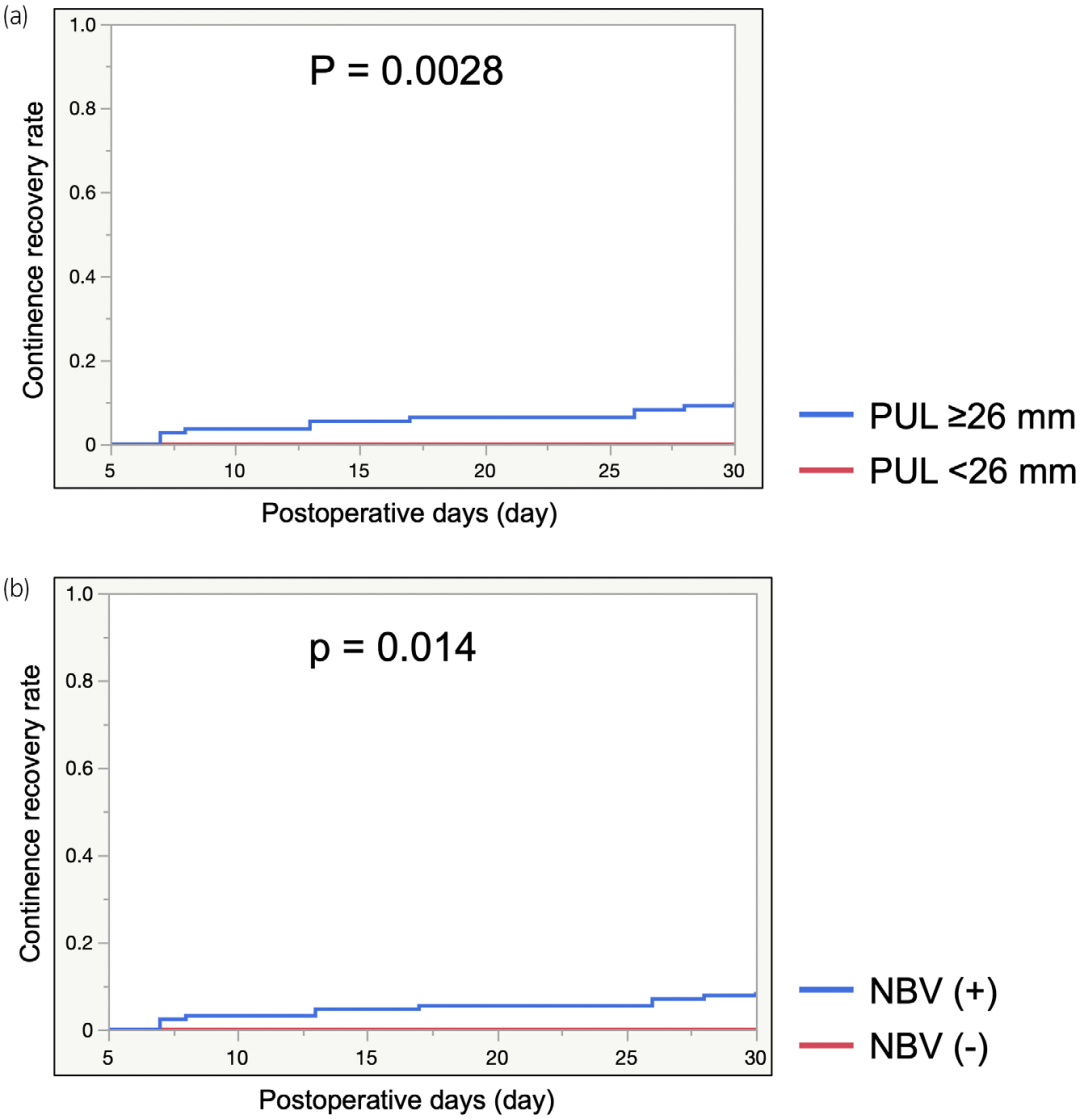
(a) Continence recovery rate curves stratified by preserved urethral length (PUL) at postoperative day 30 were plotted using the Kaplan–Meier method. A PUL ≥26 mm was a statistically significant factor that influenced urinary continence recovery at 30 days after robot‐assisted radical prostatectomy (RARP) (*p* = 0.0028). (b) Continence recovery rate curves stratified by neurovascular bundle (NVB) preservation at postoperative day 30 were plotted using the Kaplan–Meier method. NVB preservation was a statistically significant factor that influenced urinary continence recovery at 30 days after RARP (*p* = 0.014).

**FIGURE 3:**
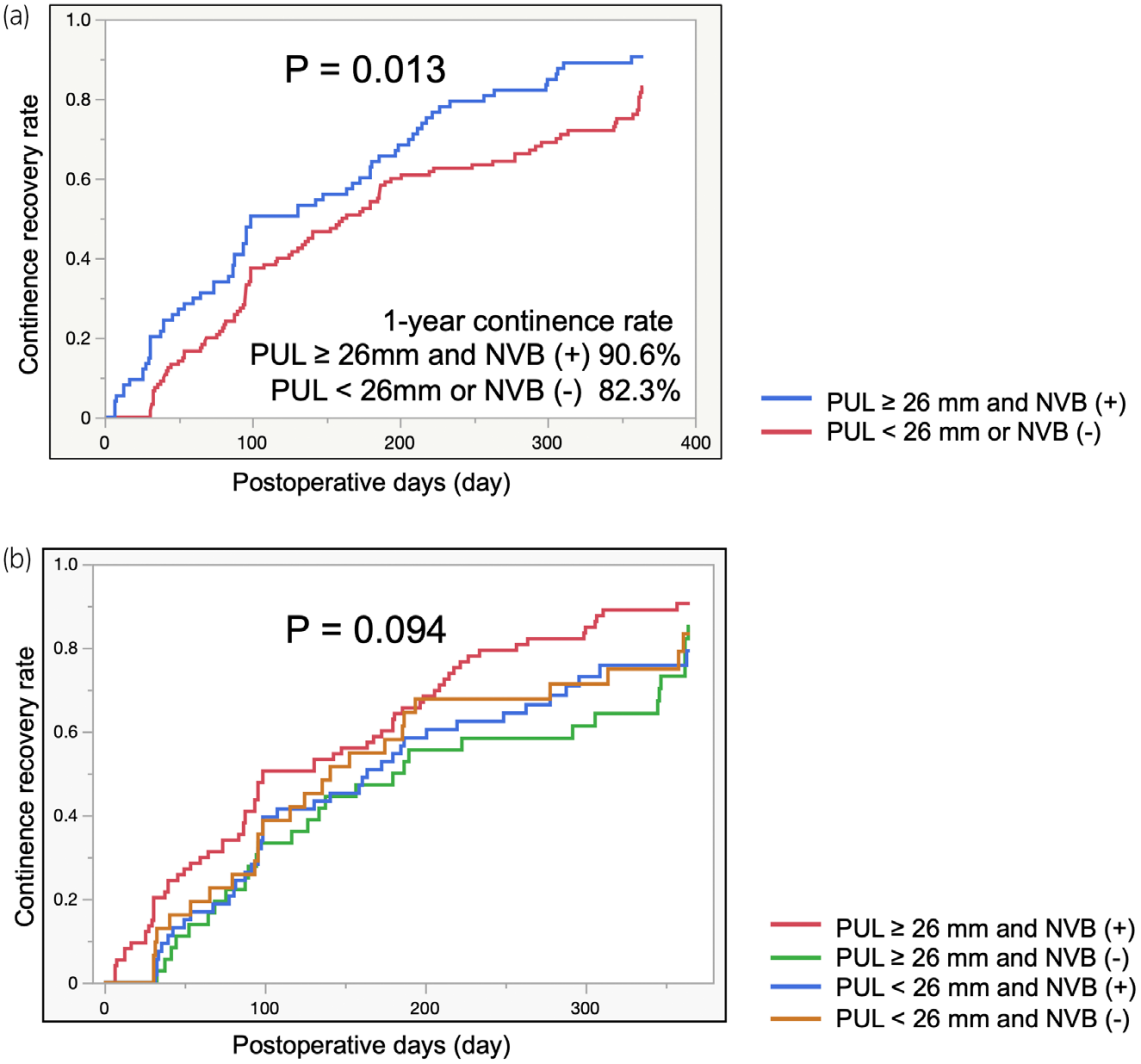
(a) Urinary continence recovery rate curves stratified by preserved urethral length (PUL) and neurovascular bundle (NVB) preservation at 365 days after robot‐assisted radical prostatectomy was plotted using the Kaplan–Meier method. A PUL ≥26 mm and NVB preservation were statistically significant factors that influenced urinary continence recovery at 365 days after robot‐assisted radical prostatectomy (RARP) (*p* < 0.013). (b) Urinary continence recovery rate curves stratified by PUL and/or NVB preservation at 365 days after RARP was plotted using the Kaplan–Meier method. Although there were no significant differences among the four groups, a PUL ≥26 mm and NVB preservation tended to have better urinary continence recovery rates than the other three groups (*p* = 0.094).

Kaplan–Meier curves for cumulative urinary continence rates showed significantly better recovery of urinary continence within 30 postoperative days in patients with a PUL ≥26 mm than in those with a PUL <26 mm (Figure 2a) and in patients with NVB preservation than in patients without NVB preservation (Figure 2b). However, significantly better urinary continence recovery within 30, 60, 90, and 365 postoperative days was observed in patients with a PUL ≥26 mm and NVB preservation than in patients without a PUL ≥26 mm or NVB preservation (Log‐rank test: *p* < 0.0001, *p* = 0.016, *p* = 0.020, *p* = 0.013, respectively) (Figure 3; Figure S6). The 1‐year urinary continence recovery rate after surgery was 87.3% for patients with a PUL ≥26 mm, 76.5% for patients with a PUL <26 mm, 86.1% for patients with NVB preservation, 84.4% for patients without NVB preservation, 90.6% for patients with a PUL ≥26 mm and NVB preservation, and 82.3% for patients with a PUL <26 mm or no NVB preservation (Figure 3; Figures S4 and S5). There was no significant difference between bilateral NVB preservation and unilateral NVB preservation in continence recovery rate (Figure S2). Among the cohort that does not include bilateral NVB preservation, there was no significant difference between the group with a PUL of ≥26 mm with unilateral NVB preservation and the other groups in continence recovery rate (Figure S3).

## DISCUSSION

In this study, we found that patients with a PUL ≥26 mm and NVB preservation had significantly better urinary continence recovery rates at 30, 60, 90, and 365 days postoperatively compared with patients with a PUL <26 mm or no NVB preservation. Although there have been some studies in the past in which NVB preservation alone or urethral preservation alone significantly improved early postoperative continence, none of those studies showed significantly better continence recovery rates after 1 year. Our results suggest that preserving both the urethra and the NVB is important in achieving urinary continence recovery.

In the patient group in the current study, all patients had a median PUL of 27 mm because the urethra was preserved to be as long as possible. The median PUL in the previous study was 10.6 mm.^6^ Although direct comparisons cannot be made because the measurement methods used in the current study were quantitative and those used in the previous study were semi‐quantitative, having a longer PUL requires actively preserving the urethra. Preoperative assessment of MUL using MRI has been reported as a possible predictor of postoperative urinary continence.^7^, ^8^, ^9^, ^10^, ^11^, ^12^, ^13^ However, even if the native membranous urethra is considered long on MRI, surgical procedures may not be able to preserve the membranous urethra length. Therefore, we considered it more important to preserve a longer urethra during the procedure than to have a longer preoperative native MUL. Our previous study measured the PUL in RARP semiquantitatively. The study showed that patients with a PUL ≥16 mm had a significantly higher rate of recovery from urinary incontinence during the overall observation period.^6^ In that previous study, the PUL varied greatly from one case to another. In another study, maximal preservation of the membranous urethra improved the continence recovery rate.^10^ Although not investigated in the present study, MUL measured on cystourethrography images at the time of removal of the postoperative balloon catheter has been reported to be the most important factor in the recovery of postoperative continence after RARP,^14^, ^15^ consistent with our findings.

Preservation of the cavernous nerve of the penis has been reported as important not only to preserve male sexual function, but also in the postoperative recovery of urinary continence. Bilateral cavernous nerve preservation is associated with increased incidence of early postoperative continence recovery after radical prostatectomy compared to unilateral nerve preservation.^16^ However, rather than nerve preservation itself, a reduction in thermal injury, increased preservation of tissue around the urethra and pelvic floor during nerve sparing, and meticulous apical dissection may improve urinary continence recovery.^16^, ^17^ Autonomic nerves from the inferior hypogastric plexus innervate the external and internal urethral sphincter and some of these autonomic fibers run within the NVBs posterolaterally, as revealed by immunohistochemistry of en bloc excised specimens of the fetal pelvis.^5^ In addition, neuroanatomical studies suggest that NVB preservation may contribute to the preservation of the autonomic nerves to the urethral sphincter, thus, improving urinary continence recovery.

When attempting to extend the PUL, attention should be paid to preventing positive surgical margins (PSMs). We compared the PSM rate in pT2 cases to our previous study and other studies. The PSM rate in patients with pT2 or less was 16.9% for all patients, 16.5% for patients with a PUL <26 mm, and 17.3% for patients with a PUL ≥26 mm at our institution. In our previous study, the PSM rate in patients with pT2 or less was 25.0% for all patients, 26.3% for patients with a PUL <16 mm, and 19.4% for patients with a PUL ≥16 mm. Thus, the PSM rate was lower in this study compared with that in our previous study. Preservation of the membranous urethra as much as possible did not increase the PSM. Other studies reported a median PSM rate of 19.0% (range, 4.7%–23.2%) with RARP in patients with pT2 or less,^18^, ^19^, ^20^, ^21^, ^22^, ^23^ suggesting that preserving the urethra was unlikely to affect PSM. However, if a preoperative MRI of the prostate, prostate needle biopsy, or digital rectal examination suggests the presence of a tumor at the apex of the prostate, attention should be paid to avoid PSM. Although data are not shown, this study includes cases of PSM located far from the apex of the prostate, which should be kept in mind when considering the PSM rate.

Many surgical techniques have been reported for achieving urinary continence after surgery. Our institution is also an educational institution for residents, and it is necessary to achieve both improvements in treatment outcomes and reduction of complications, as well as training new surgeons. An effective surgical procedure that both new and experienced surgeons can perform and that can achieve urinary continence is required. In this study, nine surgeons, including residents, performed the RARP as an operator, and, based on the cases, showed an excellent recovery rate from urinary incontinence. We believe that our surgical method is “universally designed surgery,” which can achieve an excellent recovery rate from urinary incontinence regardless of the surgeon's previous surgical experience.

Our study has several limitations. First, there are limitations inherent to the limited sample size, and performing a prospective randomized study on the advantages and disadvantages of PUL is difficult ethically. Second, this study was conducted at a single‐center, and external validation using another data set is needed. Third, the force of cephalad retraction of the prostate with a third robotic arm may result in PUL measurement error. Finally, the influence of postsurgical pelvic floor training cannot be excluded.

Our results suggest that a significantly higher postoperative urinary continence recovery rate can be achieved in patients with a PUL ≥26 mm and NVB preservation after RARP.

## AUTHOR CONTRIBUTIONS

**Satoshi Ando:** Investigation; writing—original draft; writing—review & editing; conceptualization; methodology. **Toru Sugihara:** Data curation; investigation. **Shiro Hinotsu:** Investigation; formal analysis. **Hiroto Kishino:** Writing—review & editing. **Daichi Hirata:** Writing—review & editing. **Risako Watanabe:** Writing—review & editing. **Atsushi Yanase:** Writing—review & editing. **Hirotaka Yokoyama:** Writing—review & editing. **Hayato Hoshina:** Writing—review & editing. **Kaori Endo:** Writing—review & editing. **Jun Kamei:** Writing—review & editing; supervision. **Eiichiro Takaoka:** Supervision; writing—review & editing. **Tetsuya Fujimura:** Writing—review & editing; conceptualization; supervision.

## CONFLICT OF INTEREST STATEMENT

No conflict of interest.

## APPROVAL OF THE RESEARCH PROTOCOL BY AN INSTITUTIONAL REVIEWER BOARD

This study was approved by the Jichi Medical University Ethics Committee (approval number: A19‐199).

## INFORMED CONSENT

Informed consent was taken in the opt‐out form.

## REGISTRY AND THE REGISTRATION NO. OF THE STUDY/TRIAL

Not applicable.

## ANIMAL STUDIES

Not applicable.

## Supporting information


Figure S1.

Figure S2.

Figure S3.

Figure S4.

Figure S5.

Figure S6.
