# Supplementary figures and images for: Early recovery of urinary continence after robot‐assisted radical prostatectomy is associated with membranous urethra and neurovascular bundle preservation
Source: Int J Urol. 2024 Jan 9;31(5):492–9. doi: 10.1111/iju.15388 (PMC11524102; doi:10.1111/iju.15388)

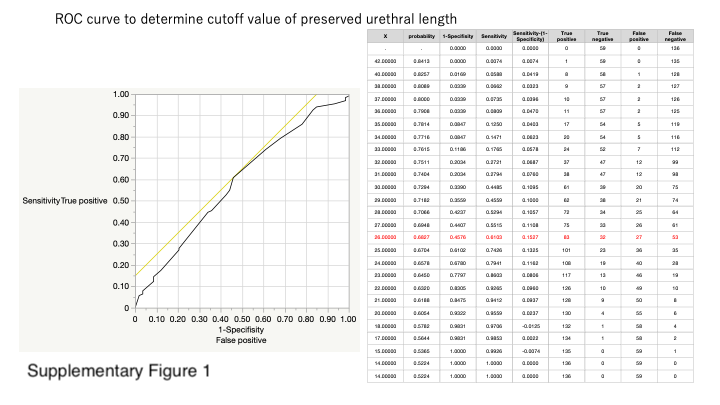

Supplement: Supplementary file 1 — Figure S1. [file IJU-31-492-s004.tiff]

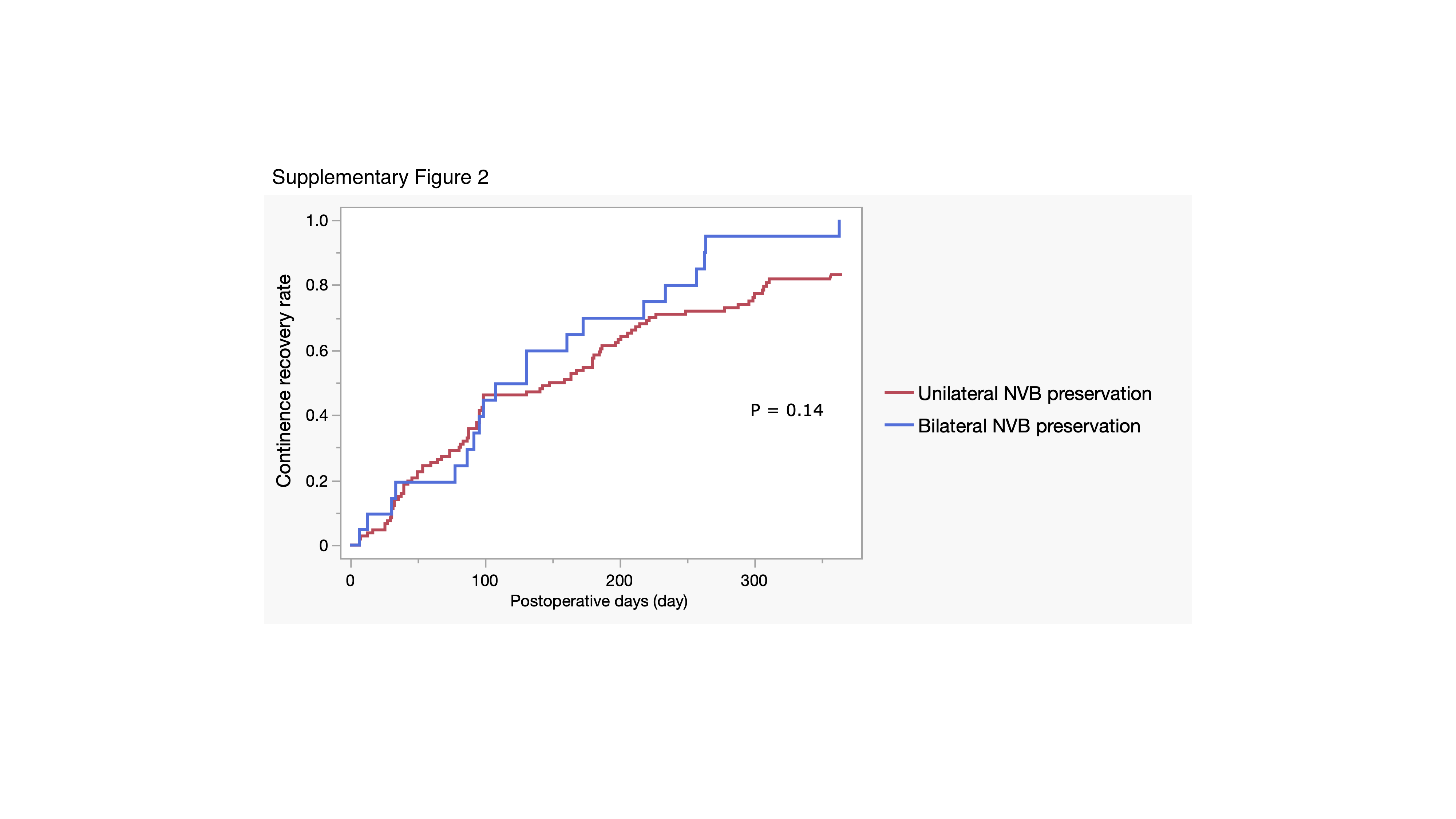

Supplement: Supplementary file 2 — Figure S2. [file IJU-31-492-s006.tiff]

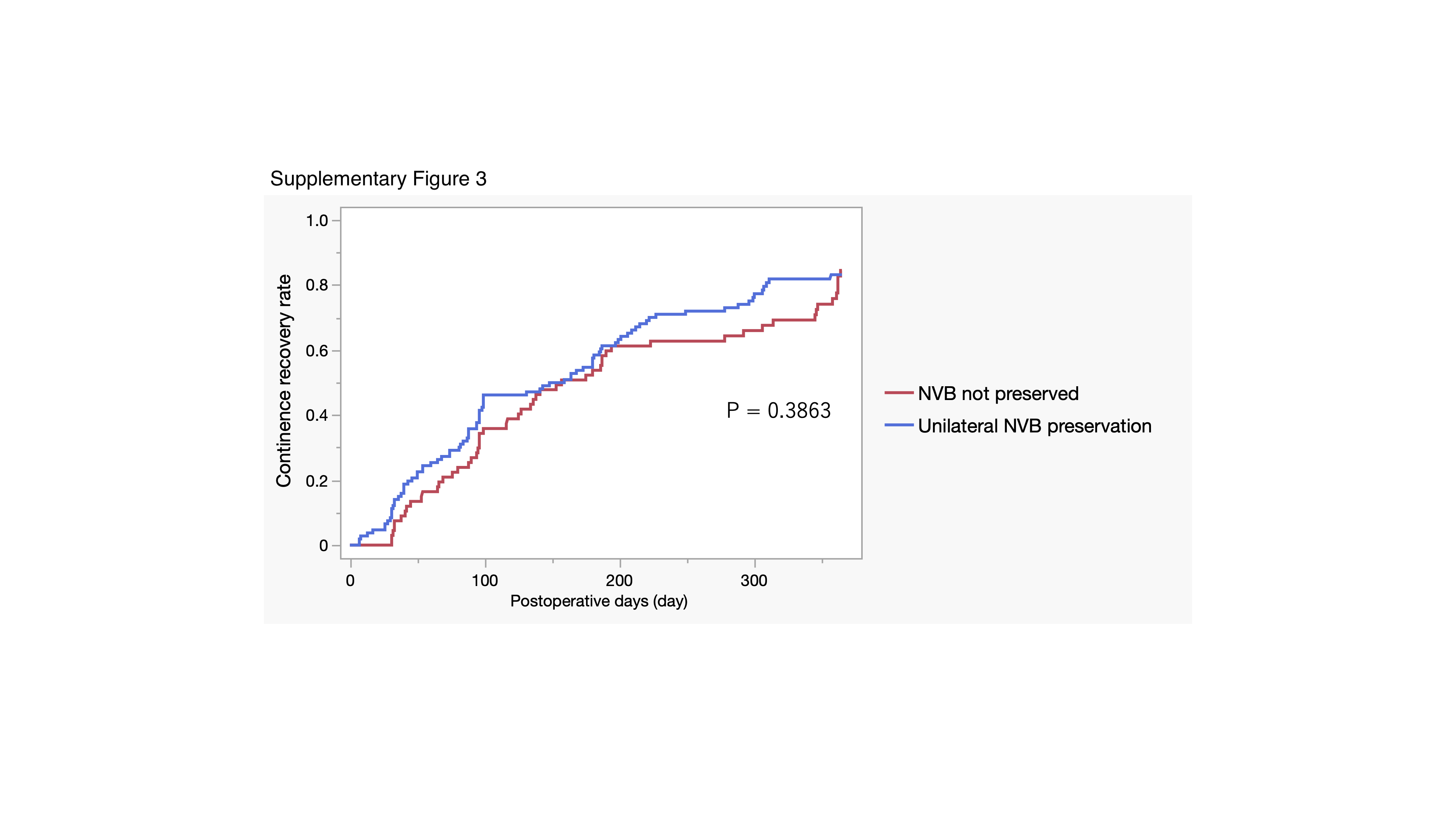

Supplement: Supplementary file 3 — Figure S3. [file IJU-31-492-s003.tiff]

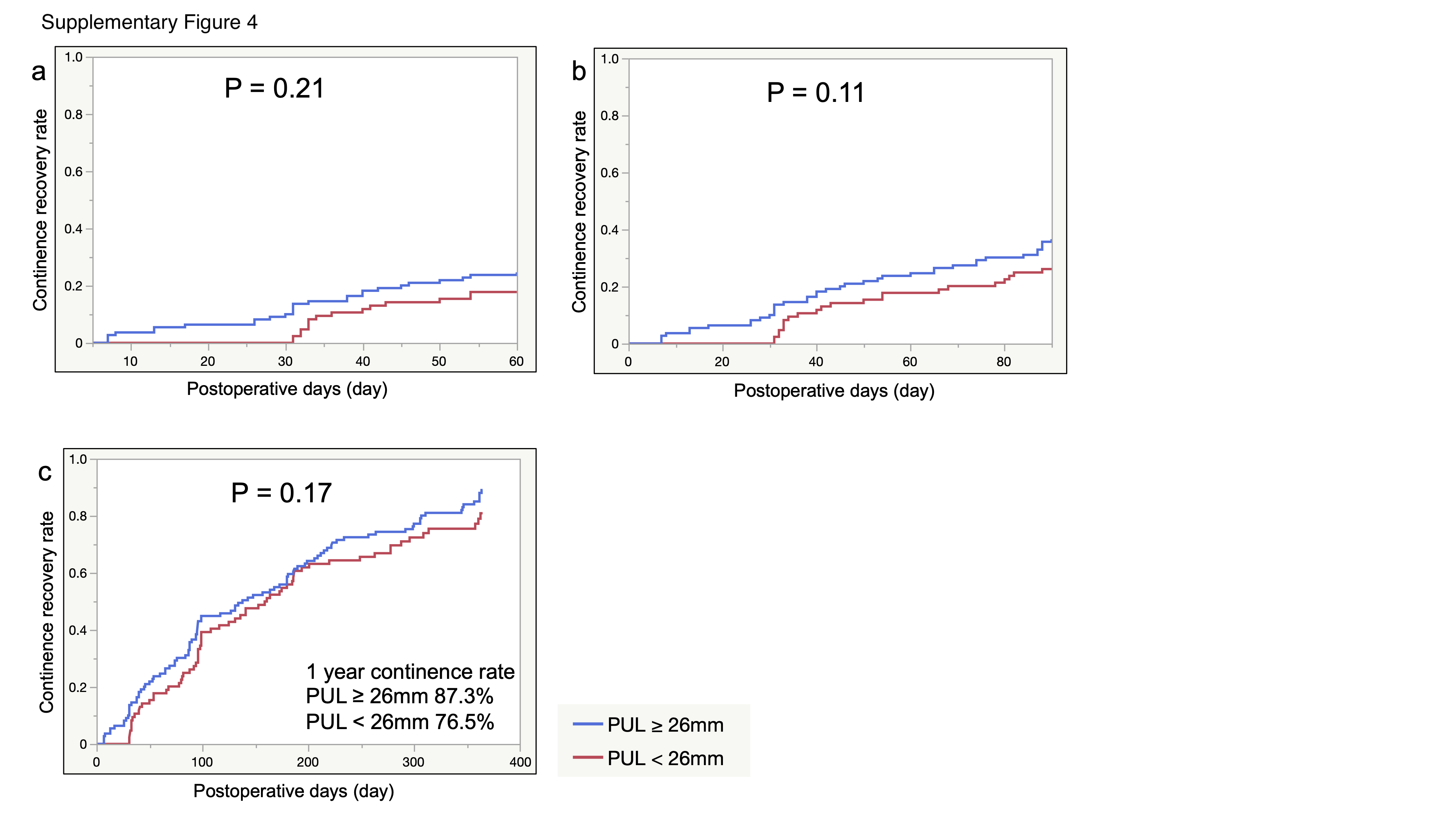

Supplement: Supplementary file 4 — Figure S4. [file IJU-31-492-s002.tiff]

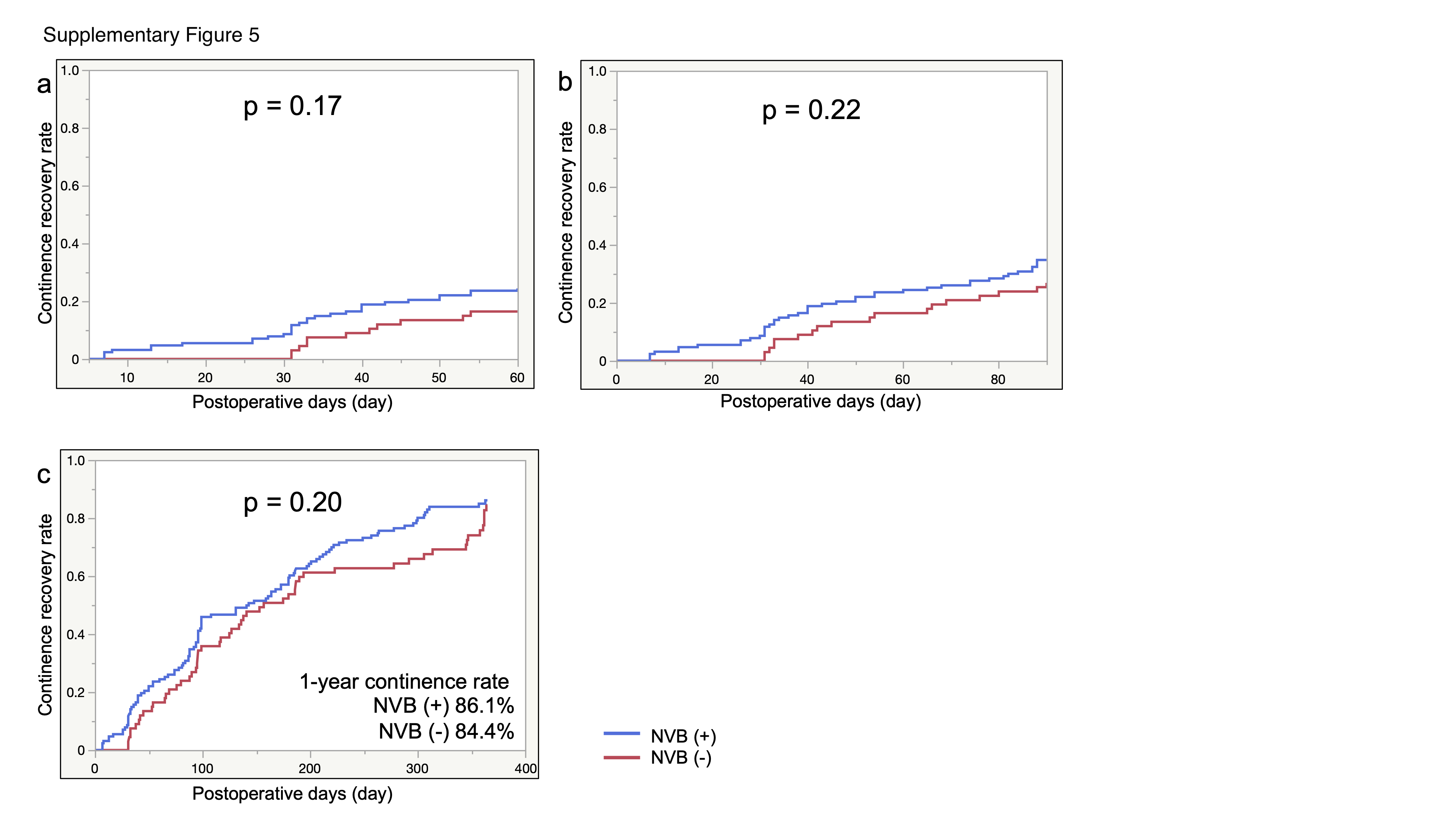

Supplement: Supplementary file 5 — Figure S5. [file IJU-31-492-s005.tiff]

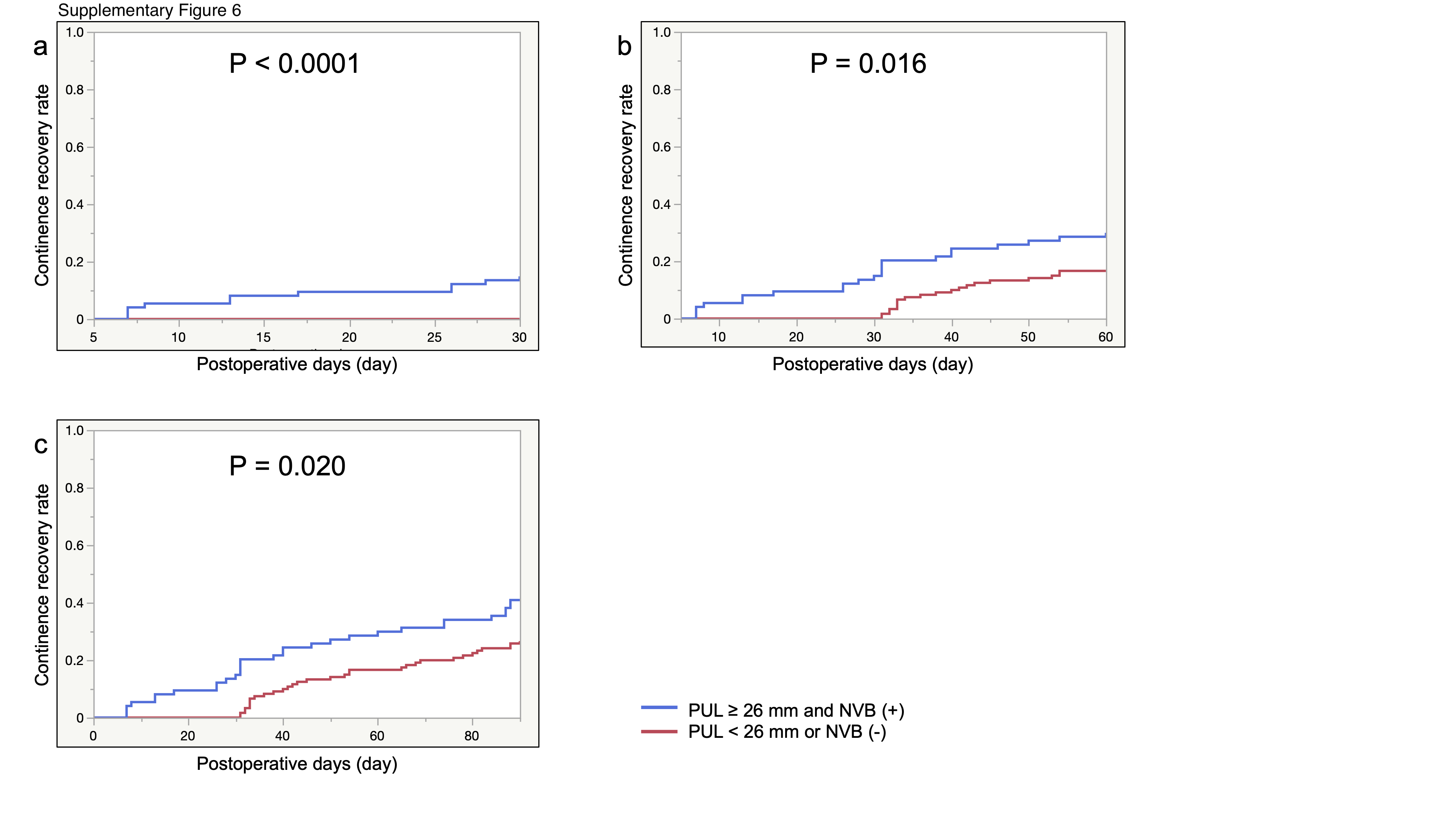

Supplement: Supplementary file 6 — Figure S6. [file IJU-31-492-s001.tiff]
